# Supplementary material for: Nanoparticle anchoring targets immune agonists to tumors enabling anti-cancer immunity without systemic toxicity
Source: Nat Commun. 2018 Jan 2;9:6. doi: 10.1038/s41467-017-02251-3 (PMC5750237; doi:10.1038/s41467-017-02251-3)
Supplement: Supplementary file 1 — Supplementary Information [file 41467_2017_2251_MOESM1_ESM.pdf]

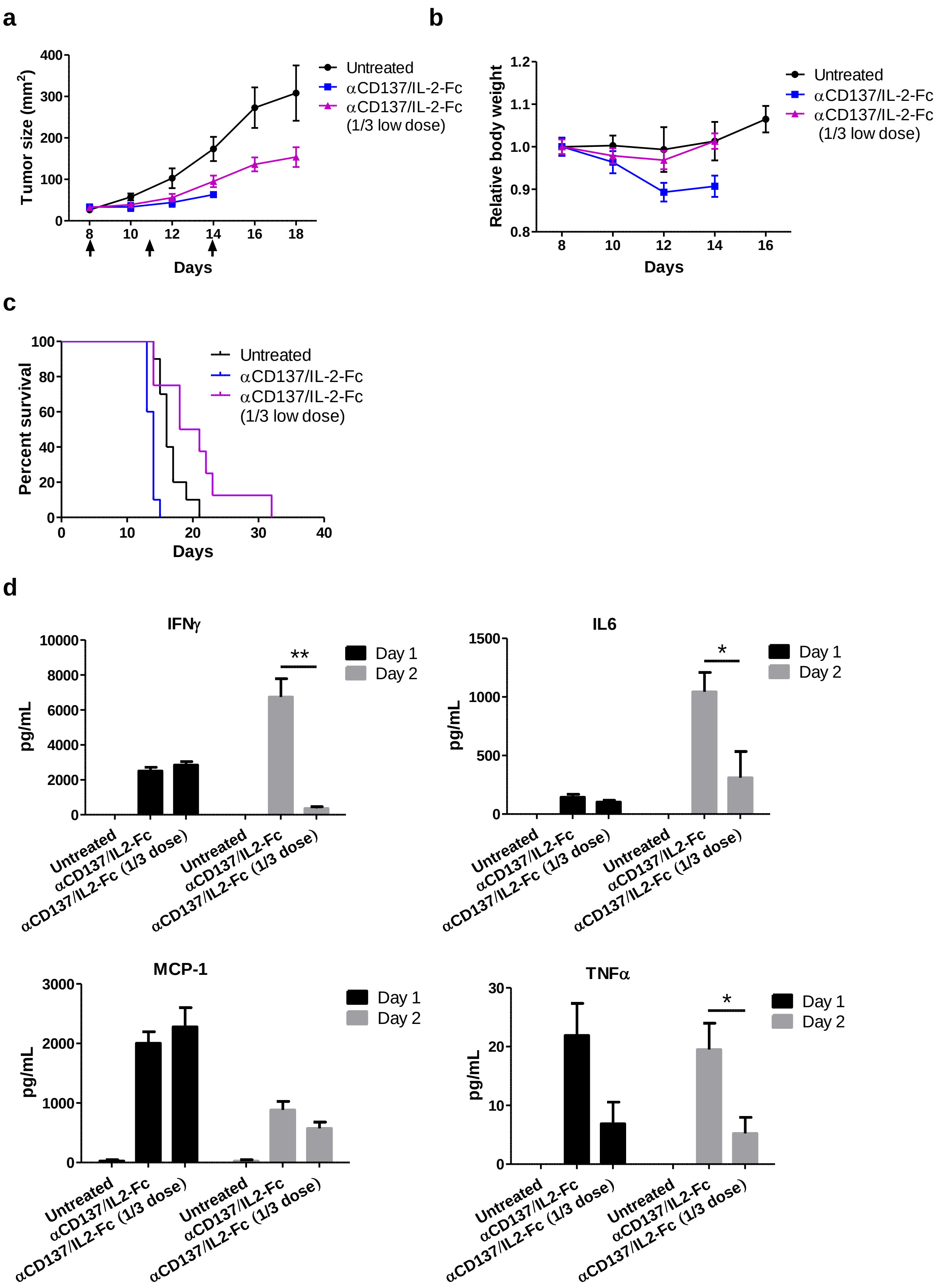
a
Tumor size (mm²)
400
300
200
100
0
8
10
12
14
16
18
Days
Untreated
αCD137/IL-2-Fc
αCD137/IL-2-Fc
(1/3 low dose)
b
Relative body weight
1.2
1.1
1.0
0.9
0.8
8
10
12
14
16
Days
Untreated
αCD137/IL-2-Fc
αCD137/IL-2-Fc
(1/3 low dose)
c
Percent survival
100
80
60
40
20
0
0
10
20
30
40
Days
Untreated
αCD137/IL-2-Fc
αCD137/IL-2-Fc
(1/3 low dose)
d
IFNγ
pg/mL
10000
8000
6000
4000
2000
0
**
Day 1
Day 2
Untreated
αCD137/IL2-Fc
αCD137/IL2-Fc (1/3 dose)
Untreated
αCD137/IL2-Fc
αCD137/IL2-Fc (1/3 dose)
IL6
pg/mL
1500
1000
500
0
*
Day 1
Day 2
Untreated
αCD137/IL2-Fc
αCD137/IL2-Fc (1/3 dose)
Untreated
αCD137/IL2-Fc
αCD137/IL2-Fc (1/3 dose)
MCP-1
pg/mL
3000
2000
1000
0
Day 1
Day 2
Untreated
αCD137/IL2-Fc
αCD137/IL2-Fc (1/3 dose)
Untreated
αCD137/IL2-Fc
αCD137/IL2-Fc (1/3 dose)
TNFα
pg/mL
30
20
10
0
*
Day 1
Day 2
Untreated
αCD137/IL2-Fc
αCD137/IL2-Fc (1/3 dose)
Untreated
αCD137/IL2-Fc
αCD137/IL2-Fc (1/3 dose)

Supplementary Figure 1: Dose reduction lowers toxicity but also lowers efficacy of IL-2-Fc/anti-CD137 combination therapy. Groups of female C57BL/6 mice (n=5/group) bearing subcutaneous melanoma as in Fig. 1 were treated with a high vs. lower dose of αCD137 (100 μg/dose *vs.* 33.3 μg/dose) and IL-2-Fc (60 μg/dose *vs*. 20 μg/dose) on days 8, 11, 14 post tumor cell inoculation. (**a**) Tumor size and (**b**) body weight changes (normalized to day 8) were monitored every other day. (**c**) Survival curve of the treatment. (**d**) One day and two days after bolus i.v. injections of high or lower doses of αCD137/IL-2-Fc, inflammatory cytokine levels in sera were measured by luminex cytokine assays. *$p<0.05$, **$p<0.0005$. All measurements shown are mean ± s.e.m.

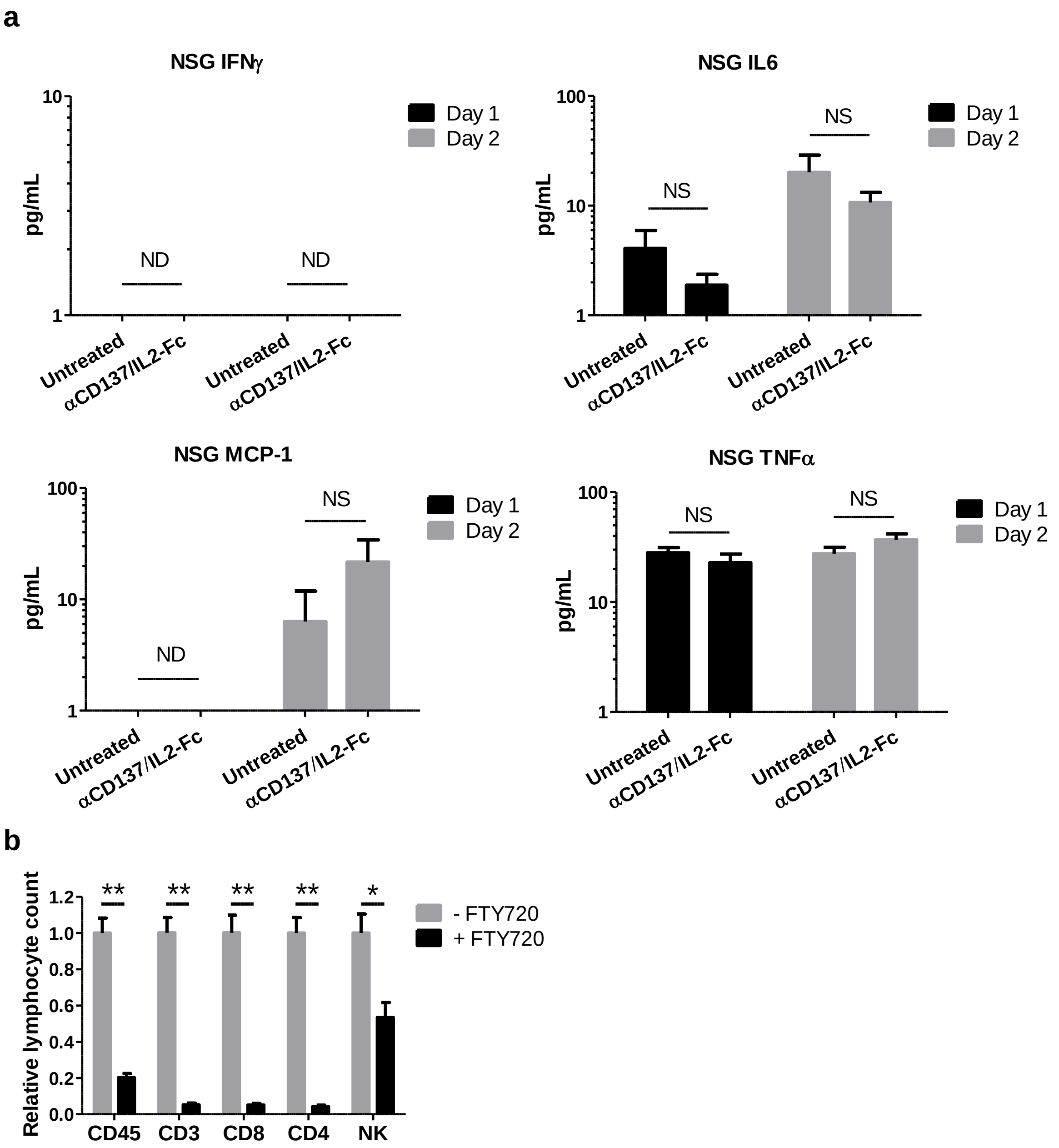

Supplementary Figure 2: Mice lacking lymphocytes or circulating lymphocytes exhibited less toxicity during IL-2-Fc/αCD137 therapy. (**a**) Groups of NSG mice (n=5/group) received i.v. injections of αCD137 (100 μg/dose) and IL-2-Fc (60 μg/dose). One day and two days later, inflammatory cytokine levels in sera were measured by luminex cytokine assays. No IFN-γ was detected at either time point. ND: not detectable; NS: not significant. (**b**) Total numbers of lymphocytes (CD45$^+$), T cells (CD3$^+$, CD8$^+$, CD4$^+$) and NK cells in peripheral blood were analyzed by flow cytometry, one day after lymphocyte depletion from circulation by intraperitoneal injections of FTY720. Results were normalized to untreated mice. *$p<0.01$, **$p<0.0001$. All measurements shown are mean ± s.e.m.

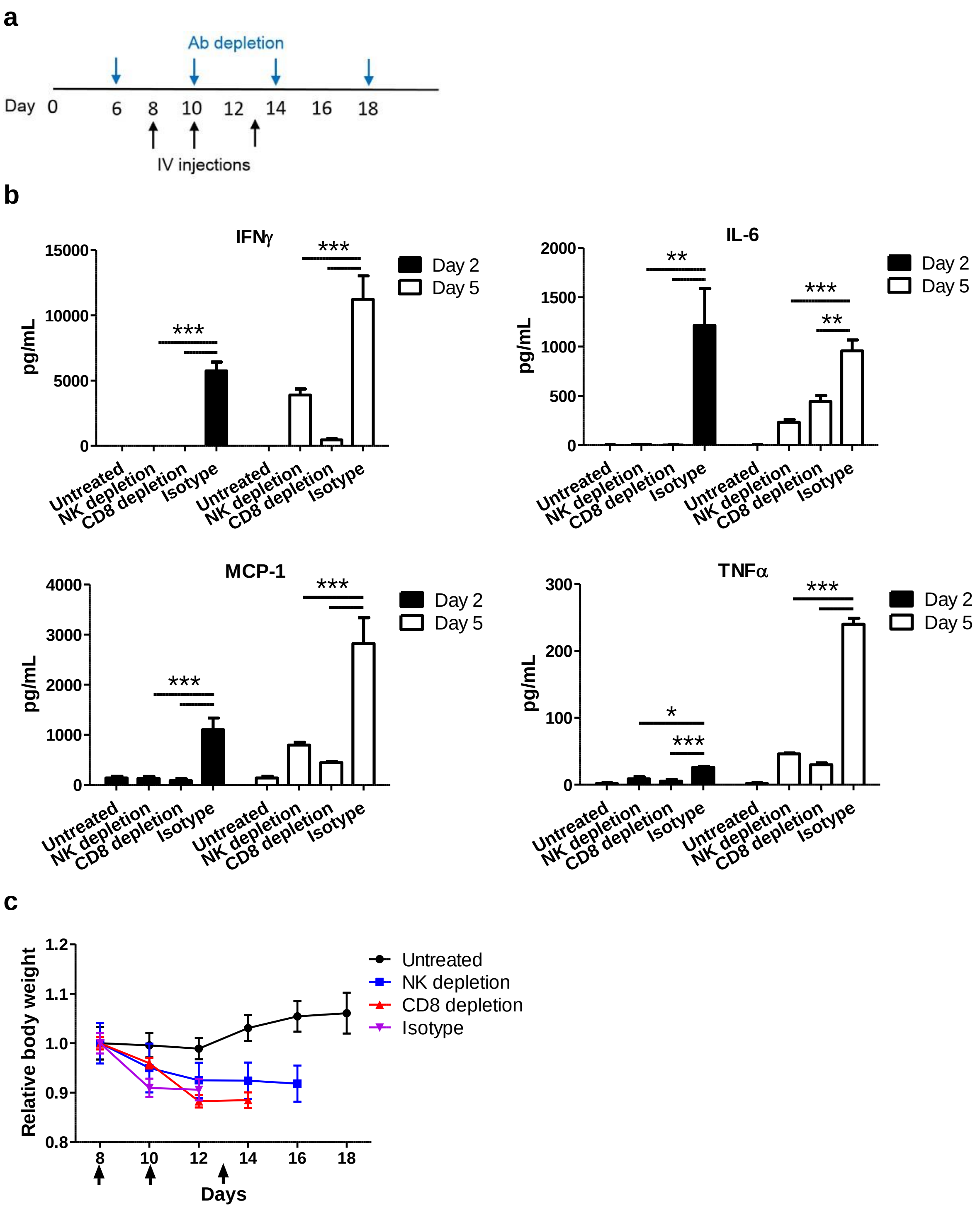

Supplementary Figure 3: NK cell and CD8^{+} T-cell depletion study. Groups of C57Bl/6 mice (n=10/group) were inoculated with 5×10^{5} B16F10 tumor cells s.c., and them received intraperitoneal injections of 400 μg NK1.1 antibody, CD8 antibody, or isotype control antibody starting on day 6, and repeated doses were given every 4 days till the end of study. Mice bearing subcutaneous melanoma received i.v. injections of αCD137+IL-2-Fc on days 8, 10 and 13 post tumor cell inoculation, with 100 μg/dose of αCD137 and 60 μg/dose of IL-2-Fc. (**a**) Timeline of antibody depletion and injections of αCD137/IL-2-Fc. (**b**) Two days after i.v. injection and five days post the first injection of the cytokine and antibody, sera from peripheral blood were collected. Inflammatory cytokine and chemokine levels in sera were analyzed by luminex cytokine assays. **p*<0.005, ***p*<0.001, ****p*<0.0001. (**c**) Relative body weight (normalized to day 8) during the study. All measurements shown are mean ± s.e.m.

a

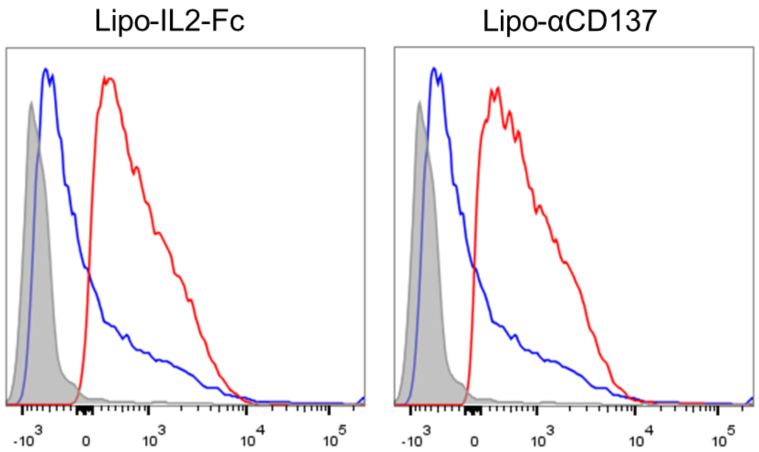

b

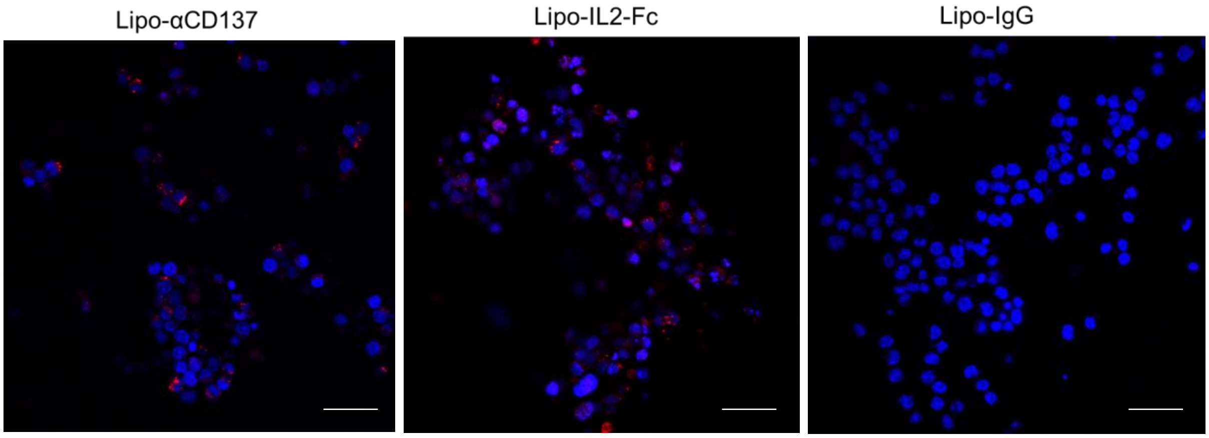

Supplementary Figure 4: Liposome-anchored immune agonists are biofunctional for T-cell binding and stimulation *in vitro*. Fluorescently-labeled Lipo-IL-2-Fc, Lipo-αCD137 or Lipo-IgG were incubated with activated $CD8^+$ T cells for 3 h. Their cellular binding to activated $CD8^+$ T cells was analyzed by (**a**) flow cytometry and (**b**) confocal microscopy. Scale bar, 20 µm. For flow cytometry plots, grey: untreated; blue: Lipo-IgG; red: Lipo-αCD137 or Lipo-IL-2-Fc. For confocal images, red: immunoliposomes; blue: nuclei.

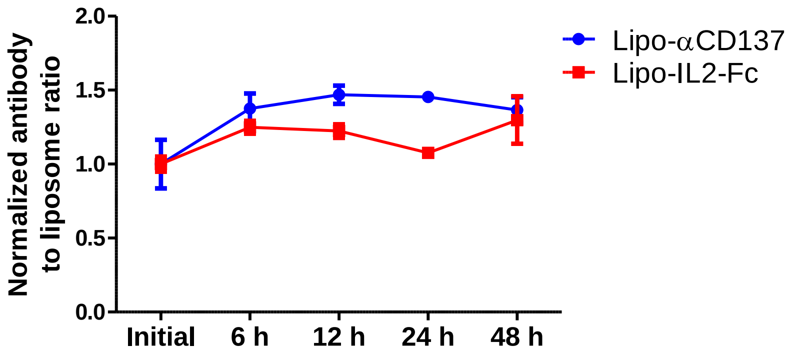

Supplementary Figure 5: Anti-CD137 and IL-2-Fc stably conjugated on the liposome surface. Alexa Fluor 488-labeled OVA was encapsulated in liposomes as a tracer of the intact vesicles, and then IRDye 800CW-labeled anti-CD137 or IL-2-Fc were conjugated to these OVA-carrying vesicles. The ratios of anti-CD137 or IL-2-Fc to liposomes (OVA signal) over time were measured *in vitro* at 37℃ with continuous rotating, using PBS containing 10% FBS as a release medium. At predetermined time points, the release medium containing fluorescence labeled immunoliposomes was subjected to ultracentrifuge at 50,000 rpm at 4℃ for 4 h. The fluorescence intensities of Alexa Fluor 488 and IRDye 800CW in the immunoliposome pellets were measured by plate reader and LI-COR, respectively. Normalized to the initial time point (set ratio as 1), the relative ratios of anti-CD137 or IL-2-Fc to liposomes were calculated. Measurements shown are mean ± s.e.m.

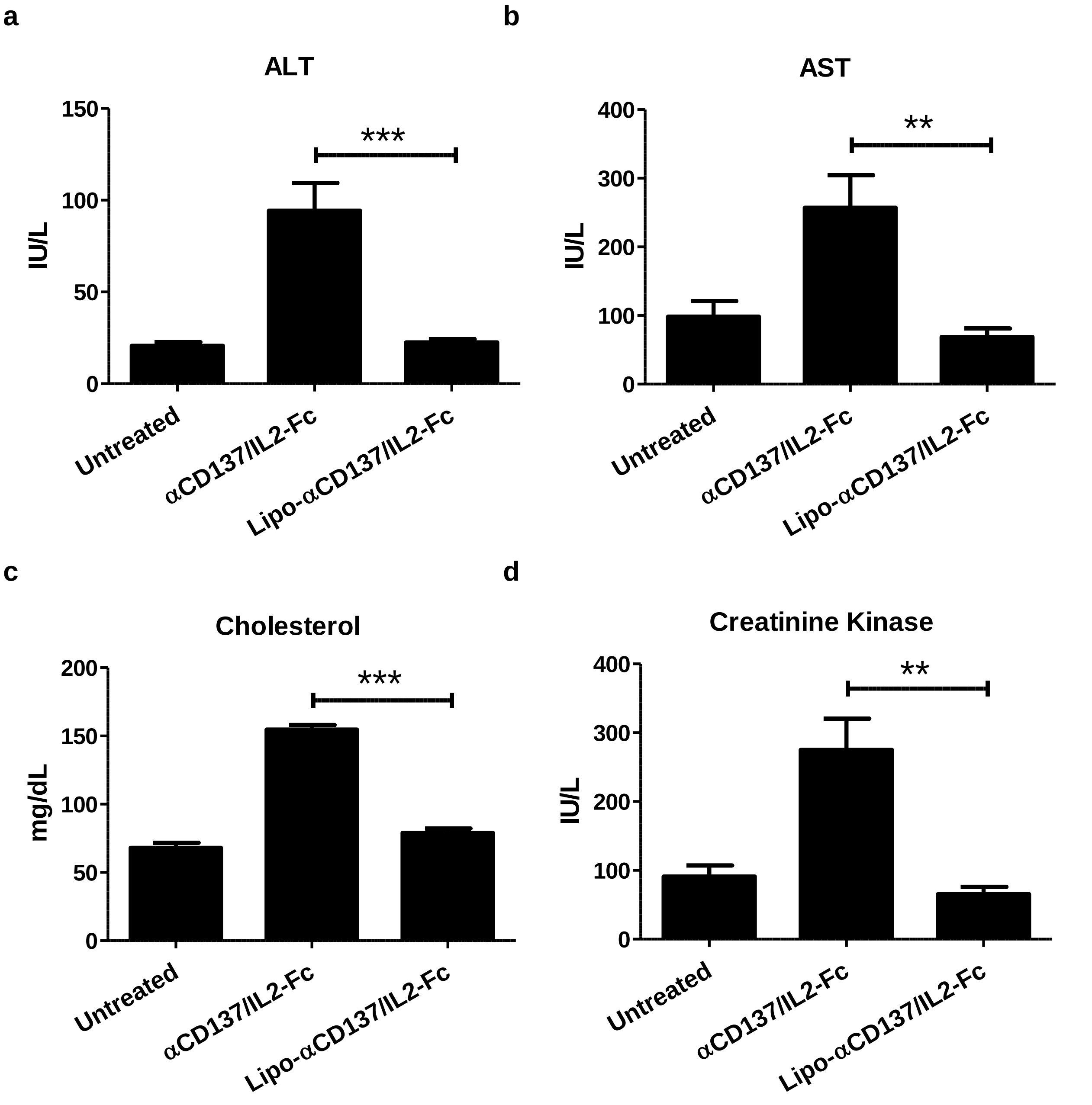

Supplementary Figure 6: Liposome delivery abrogates signatures of liver and kidney toxicity induced by αCD137/IL-2-Fc therapy. Groups of C57Bl/6 mice (*n*=6/group) were inoculated with 5×10^5 B16F10 tumor cells s.c. on day 0, then received i.v. injections of αCD137 + IL-2-Fc or Lipo-IL-2-Fc + Lipo-αCD137 on day 8 and 10. On day 11, peripheral blood samples were collected for analysis of serum levels of alanine transaminase (ALT, **a**), aspartate transaminase (AST, **b**), cholesterol (**c**), and creatinine kinase (**d**). $^{**}p<0.005$, $^{***}p<0.001$. All measurements shown are mean ± s.e.m.

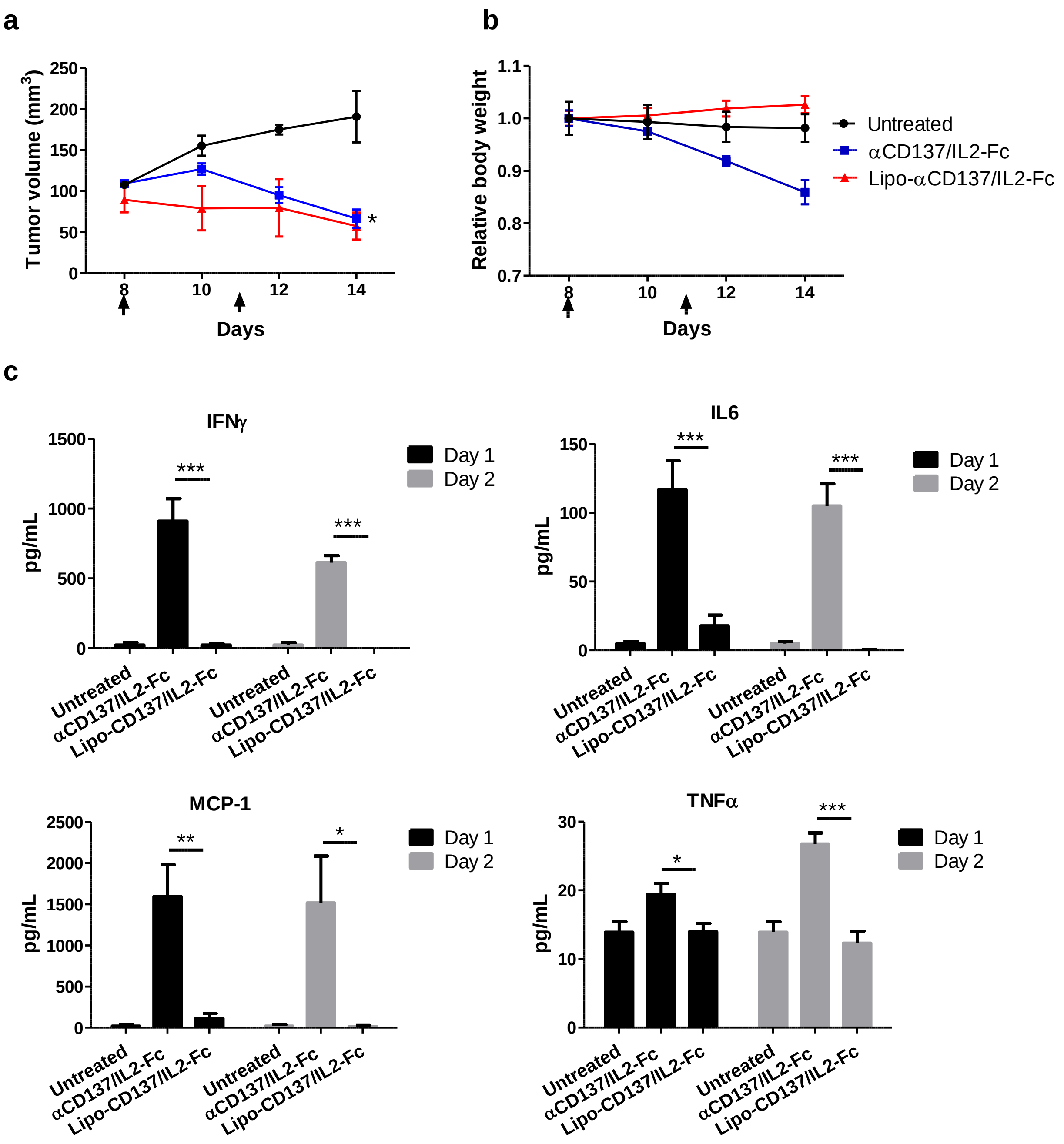

Supplemental Figure 7: Immunoliposomes suppress tumor growth in the A20 B cell lymphoma model without toxicity. Groups of balb/c mice (n=5/group) were injected with 2×10^6 A20 cells s.c. on day 0. Untreated animals were compared to mice that received i.v. injections of αCD137 + IL-2-Fc or Lipo-αCD137 + Lipo-IL-2-Fc on days 8 and 11 after tumor cell inoculation, (100 µg/dose αCD137 and 60 µg/dose IL-2-Fc). (**a**) Tumor growth vs. time. *$p<$ 0.0005, Lipo-CD137/IL-2-Fc vs. Untreated. (**b**) Relative body weights during the therapy (normalized to day 8). (**c**) One day and two days after i.v. injections, sera from peripheral blood were collected, and inflammatory cytokine/chemokine levels in sera were analyzed by luminex assays. *p<0.05, **p<0.005, ***p<0.001. Shown are mean ± s.e.m.

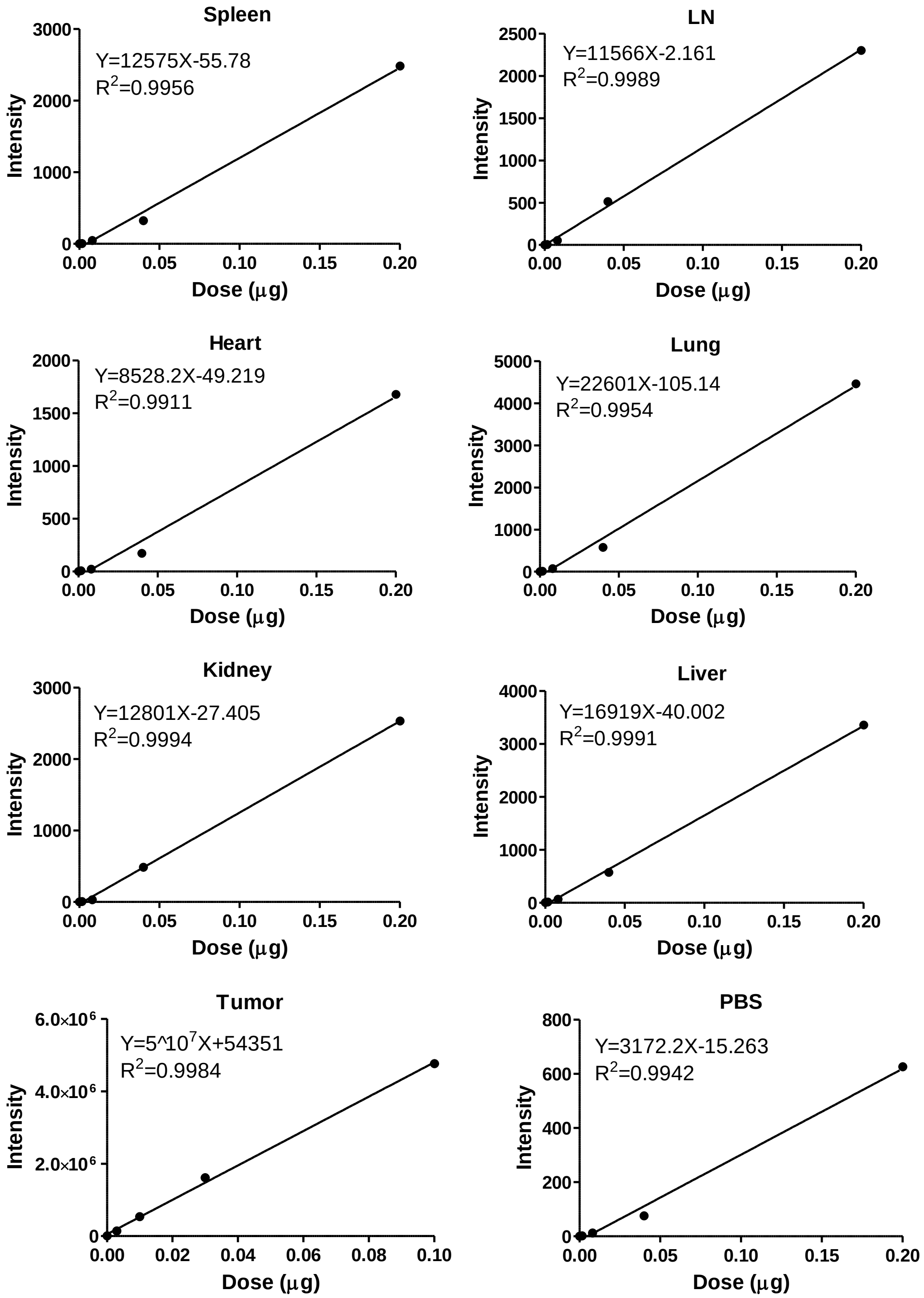

Supplemental Figure 8: Standard curves of IRDye CW800 in organs and PBS. Organs were dissected from mice and known doses (0, 0.0016, 0.008, 0.04, 0.2 μg) of IRDye CW800 infrared dye were injected into individual organs before the fluorescence intensities were measured using a LI-COR Odyssey imaging system. The standard curve equations and R-squared (R^2) values were determined by Excel and GraphPad Prism software.
